# Infants’ Looking to Surprising Events: When Eye-Tracking Reveals More than Looking Time

**DOI:** 10.1371/journal.pone.0164277

**Published:** 2016-12-07

**Authors:** H. Henny Yeung, Stephanie Denison, Scott P. Johnson

**Affiliations:** 1 Department of Linguistics, Simon Fraser University, Burnaby, British Columbia, Canada; 2 Department of Psychology, University of Waterloo, Waterloo, Ontario, Canada; 3 Department of Psychology, University of California Los Angeles, Los Angeles, California, United States of America; Medical Photonics Research Center, Hamamatsu University School of Medicine, JAPAN

## Abstract

Research on infants’ reasoning abilities often rely on looking times, which are longer to surprising and unexpected visual scenes compared to unsurprising and expected ones. Few researchers have examined more precise visual scanning patterns in these scenes, and so, here, we recorded 8- to 11-month-olds’ gaze with an eye tracker as we presented a sampling event whose outcome was either surprising, neutral, or unsurprising: A red (or yellow) ball was drawn from one of three visible containers populated 0%, 50%, or 100% with identically colored balls. When measuring looking time to the whole scene, infants were insensitive to the likelihood of the sampling event, replicating failures in similar paradigms. Nevertheless, a new analysis of visual scanning showed that infants *did* spend more time fixating specific areas-of-interest as a function of the event likelihood. The drawn ball and its associated container attracted more looking than the other containers in the 0% condition, but this pattern was weaker in the 50% condition, and even less strong in the 100% condition. Results suggest that measuring where infants look may be more sensitive than simply how much looking there is to the whole scene. The advantages of eye tracking measures over traditional looking measures are discussed.

## Introduction

Since at least the early 1960’s, looking time (LT), typically measured by recording cumulative gaze to a whole visual scene, has been one of the most commonly used behavioral indices of infants’ cognitive and perceptual capabilities [1–3]. Recently, however, there has been increasing acknowledgement that traditional LT measures are limited [4], especially in comparison to other, more detailed measures of eye gaze [5,6]. Increasingly, both infant and adult research studies measure other types of gaze information, asking how looking to specific locations reveal more subtle measures of cognitive ability. Such techniques have been used to explore implicit theory-of-mind [7–10], perceptual categorization abilities [11–15], as well as many other aspects of cognitive and perceptual processing (e.g., [16–20]). In the present report, we focus on a research domain in which these advanced eye-tracking techniques have not yet been applied: Infants’ understanding of surprising sampling events in procedures that measure inferential reasoning, and which have garnered significant attention in the cognitive development literature in recent years [21–23].

We implemented a novel sampling paradigm, which involved either surprising, neutral, or unsurprising events: A single ball was drawn from boxes populated with 0%, 50%, or 100% identically colored balls, respectively. The critical events (0% versus 100%) were deterministic, and thus maximized the contrast between surprising and unsurprising events, at least relative to comparable reports that manipulated the probability of sampling (e.g., 25% versus 75%), which have not always found the predicted pattern of a violation of expectation [24,25]. We replicated this failure to find clear LT patterns (even with more contrastive, deterministic sampling), but our results also showed a novel finding. More sophisticated measure of visual scanning (gaze to different locations) revealed sensitivity to the relative surprise of the events, undetectable with standard LTs.

Below, we review previous work on sampling events as a measure of inferential reasoning, and then present the motivation for the current study. We conclude by arguing that measures of visual scanning (and not just gaze to the whole scene) may be better able to probe infants’ reasoning capabilities than traditional LT measures.

### Sampling events as a measure of inferential reasoning

Humans often reason about uncertain events, and the beginnings of such inferential reasoning processes seem to emerge in infancy, as reported in studies that observed longer LTs to sampling events that are statistically more improbable than probable [7,8]. These findings have been replicated using a variety of methods, including many LT-based procedures [23,24,26–29], crawling and searching procedures [30,31], as well as a procedure assessing the timing of infants’ object manipulation [32].

In a typical LT sampling procedure, infants see a container filled with a certain proportion of two object types (e.g., red and yellow balls). This container is then randomly sampled, as one (in a single-event sampling procedure) or several objects (in a multi-event sampling procedure) exit the container. Previous results have documented infants’ sensitivity to a number of factors influencing event probabilities, including the physical properties of the objects [27,28] or containers themselves [22], the location of the objects within the containers [23,24], and the psychological preferences on the part of the person sampling [15].

Recent findings highlight important limitations in inferential reasoning, particularly about inferences made from single events (i.e., the sampling of a single object). In one such report, which recorded LTs, 12-month-old infants failed to make sampling inferences when a population contained more objects than can be tracked (e.g., a 12-to-4 population of blue to yellow items), but succeeded with smaller numbers in the same ratio (e.g., a 3-to-1 population). The authors interpreted this result as suggesting that distinct cognitive systems for small and large numerosities interact with probabilistic reasoning abilities [25]. Similarly, in a second looking-time task, 12-month-olds were insensitive to sampling probability with a 6-to-1 population (and instead focused on the perceptual features of the scene), but *were* sensitive to sampling probability with a 4-to-1 population (10). In this same task, 8-month-olds appeared to only be sensitive to perceptual features, and so the authors concluded that probabilistic inference with single events undergoes significant development between 8 and 12 months.

Curiously, findings from these two looking-time tasks conflict with other reports showing success in 10- to 12-month-olds at reasoning about single samples from large collections of objects. One difference is that these latter experiments used an explicit choice task (i.e., crawling and searching) when testing samples drawn from large collections of objects ([16,17]; see [19] for similar findings with apes). In sum, LT experiments on infants’ reasoning abilities have revealed some failures to make inferences about single samples from distributions containing more items than can be individually tracked, but other tasks have revealed success.

### The current study

The above discrepancies in the literature largely motivate the present methodological investigation, where we measured both a) LTs and b) infants’ visual scanning after presenting single sampling events from three large distributions. Because this is the first use of an eye tracker to scan sampling events, we intentionally presented events contrasted as perspicuously as possible (0% vs. 50% vs. 100%), and thus predictions about the sampling event (e.g., one red ball draw) were either deterministic in nature (one container held only red balls, another only yellow balls) or statistically neutral (another container held 50% red and 50% yellow balls). While our results cannot specifically address outstanding questions about task-dependent discrepancies in the larger literature on infants’ probabilistic reasoning, the present report is a necessary first step in addressing that debate, and has broader methodological implications for the wider field.

To our knowledge, only one published study has reported the location of infant gaze when observing a sampling event [12]. An experimenter was seen manipulating two boxes containing complementary 4:1 ratios of pink and yellow balls. Critically, the authors analyzed *which* box infants fixated during and after sampling (the one from which the sample was drawn or the other one). No clear patterns were found with respect to whether gaze predicted individual infants’ understanding of the sampling, but their analysis was intended to control for alternative interpretations of their main finding. In fact, their analysis could not show whether infants recognized something unexpected about the sampled container when it yielded an improbable outcome.

Our prediction was that infants should devote increased attention to a sampled container (and the sample itself) after an improbable event. To assess this possibility, we developed a novel eye-tracking procedure. First, to control for possible differences in attractiveness, all containers remained covered after sampling. This change also meant that gaze could not be influenced by perceptual comparisons between the sample and the container (unlike the procedure in [12]). Second, we introduced three containers in order to increase the number of target locations at which to gaze. Third, we used containers that had clearly distinct proportions (either 0%, 50%, or 100% of one color), which maximized the contrast between containers. Finally, we used a single ball draw from large collections of balls, and tested 8- to 11-month-old infants in order to directly address the above-discussed discrepant findings on single-event probabilities in a comparable age range.

## Method

### Participants

Consent procedures were approved by the UCLA North General Institutional Review Board, which included written consent from all infants’ parents prior to study participation. The study was also halted if infants showed any signs of not assenting to participate in this research (by crying or fussing for an extended period of time).

Twenty-six infants participated in this study, but data from 7 infants were excluded because no eye-tracking data was collected in at least one test trial. In all cases, this was because infants were fussy, crying, or otherwise disinterested in the visual events of that trial. The remaining 19 infants (10 male; *Mean* = 9.5 months; *Range* = 7.7 months—11.1 months) were full-term, and heard mostly English in the home (> 50%).

### Stimuli

#### Physical stimuli

We constructed several transparent Plexiglas containers in order to demonstrate the sampling events. The three largest containers had a green felt cover, which concealed the contents of the container. All of these large containers had 36 Ping-Pong balls that were either red or yellow: One contained 0 red balls (0%), another had 18 red balls (50%), and the last had 36 red balls (100%). When the green felt cover was pulled back, roughly 15–20 red and yellow balls were visible, and in the 50% container, the distribution of red and yellow balls was relatively uniform (i.e., they were not clumped together).

#### Videos of physical stimuli

**Fig 1:**
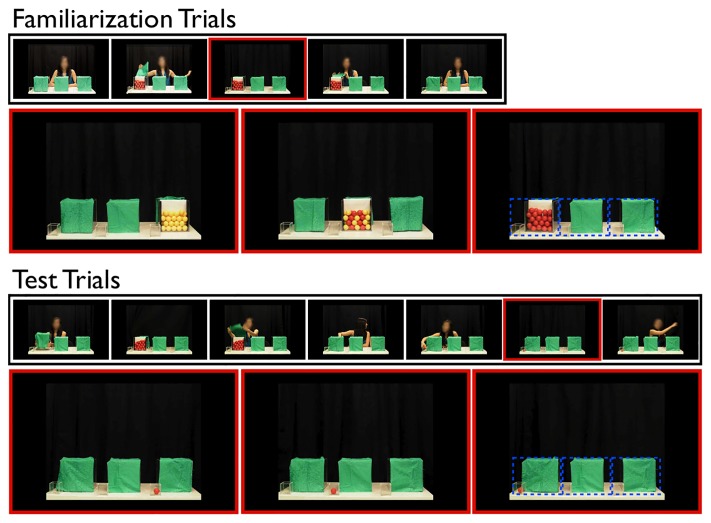
Example video frames from the experimental procedure. Images outlined in red are video frames where playback froze, and was contingent upon infant looking. The top section shows a 5-image sequence from a familiarization trial, and three examples of critical video frames from those trials in the 0%, 50%, and 100% sampling conditions (left to right, assuming a red ball draw). The bottom section shows a 7-image sequence from a test trial, and three examples of critical video frames from those trials in the 0%, 50%, and 100% sampling conditions. Example areas-of-interest (AOIs) are indicated by the dashed boxes (in blue). The face of the woman in the video is blurred only for the purposes of publication, and was not blurred in the videos presented to infants.

Two introductory videos, three familiarization videos, and three test videos were filmed, and the staging was intended to mimic infant-controlled looking procedures testing probabilistic inference (e.g., [7]). All videos featured a woman at a table, which was in front of a black curtain (Fig 1). In the first introductory video (32 s), the woman manipulated two red and two yellow balls in an oblong transparent container, while speaking short attention-getting phrases every few seconds (“Look at this one!” “Watch this!” “Wow!”). In the second introductory video (21 s), three small, cubical transparent containers (that could hold a single ball) were seen on the table. One-by-one, the woman said, “Hey, look at this!” and then placed each of three large containers covered in green felt next to each small container. All six containers remained in place in subsequent videos.

In each of the familiarization videos, the woman began by saying, “Hey, look at this!” She then grabbed one of the three large containers, shook it, and then placed it back on the table. She then pulled back its green felt cover, which exposed the contents of this container, said, “Look!” and then hid behind the curtain (12 s). At this point, the video froze, and infant looking time was recorded (see Procedure). The video ended when the woman reappeared from behind the curtain, re-covered the target container and said, “Good job!” (8 s).

In each of the test videos, the woman shook one large container, placed it back down, said, “Look!” and uncovered it, and then hid herself again behind the curtain (about 10 s). After a fixed amount of time (5 s), she reappeared, re-covered the target container, and then said, “Watch this!” (about 5 s). She then looked away while retrieving one ball from the target container with her hand. Then she said, “Look!” and placed the drawn ball into the small transparent container next to the target large container and hid herself behind the curtain (about 5 s), after which point the video froze and looking time was recorded until the infant looked away for 2 consecutive seconds. These videos ended as the woman reappeared and said, “Good job!” (5 s). Samples of all the original stimulus videos are available from the authors upon request.

### Procedure

Infants were tested in a softly lit room booth with black curtains, sitting on their parent’s lap about ~ 55 cm from a SR EyeLink 1000 (sampling rate: 500 Hz). A camera located off to the side recorded the infant’s behavior, which was visible to an experimenter who did not know the order of trials, and could not see the stimulus display. Parents were asked to remain silent, not look at the screen, and not interact with their baby when stimuli were presented.

Infants were shown 14 trials, each of which consisted of the presentation of one stimulus video. Short animations were displayed between trials to maintain infants’ attention. The experimenter coded looking time at critical moments by pressing a button on a keyboard (see below), and the eye tracker also recorded the gaze of the infant. In the first 2 trials, the two introductory videos were shown in their entirety. The first trial showed the experimenter manipulating Ping-Pong balls, which were shown to be freely movable, solid objects, and the second trial introduced the 3 pairs of containers being positioned on the left, center, and right of the screen.

The next 6 trials familiarized infants with the contents of the large containers. The three familiarization videos were presented in an infant-controlled manner: After the contents of the target container were revealed in the video, playback froze until infants looked away for 2 consecutive seconds. This was coded online by the experimenter in order to maintain a similar protocol as previous paradigms investigating infant reasoning abilities [21,24,25], but it should be noted that gaze contingency can also be measured using eye tracker data, which can yield more accurate infant-controlled designs, particularly at younger ages [33,34]. Once infants looked away, playback restarted (typically recapturing infants’ attention), and ran until the end of the video. A sequence of 3 videos presenting each of the familiarization trials was repeated once.

In the last 6 trials, each of the three test videos was also presented in an infant-controlled manner: Playback froze after a ball was drawn and once the woman had hid herself (see Stimuli description above), and restarted for the next trial when the infant looked away for 2 consecutive seconds. These trials measured infants’ response to a single sampling event. After each of the 3 videos were presented once, they were shown again in the same order.

Critically, in familiarization and test trials, one large container was always the 0% one, one large container was always the 50% one, and the last was always the 100% one. The positions of the containers and the color of the drawn ball were fixed within subjects, and the 50% container was always in the center of the table. However, the positions of the 0% red and 100% red containers, and the color of the drawn ball were counterbalanced across infants. In addition, the order of trials during familiarization and test phases (i.e., whether the 0%, 50%, or 100% container was manipulated first) was also fixed within subjects, and counterbalanced across infants.

## Results

In order to compare our results with previous LT methods, we analyzed eye tracker data from the critical portions of familiarization and test trials (when stimulus playback froze after the ball was drawn, a period that was contingent upon infant gaze as coded by the experimenter). This was done to eliminate the possibility that infants would look at the actor (who was hidden when stimulus playback froze), and to make our procedure as similar as possible to paradigms using traditional LT measures (e.g., [21]), which typically measure LTs just after the sampling event, once the actor conceals herself (or lowers her head).

We analyzed two principal types of measures: First, accumulated samples of infants’ gaze to the overall scene were collected from the eye tracker (sampling rate: 500 Hz), experimenter-coded LTs of the same variable were also calculated. Second, we also analyzed total gaze (accumulated fixations, defined as the point of gaze remaining in within .5° for at least 100 ms) in three distinct areas of interest (AOIs) that contained associated pairs of small and large containers. These AOIs are indicated in Fig 1. Our main question was whether LT towards the whole scene, or to the containers (the AOIs) varied as a function of ball-draw likelihood. Data are accessible at http://researchdata.sfu.ca/islandora/object/islandora:495.

### Infants’ gaze to the whole scene

#### Familiarization trials

**Fig 2:**
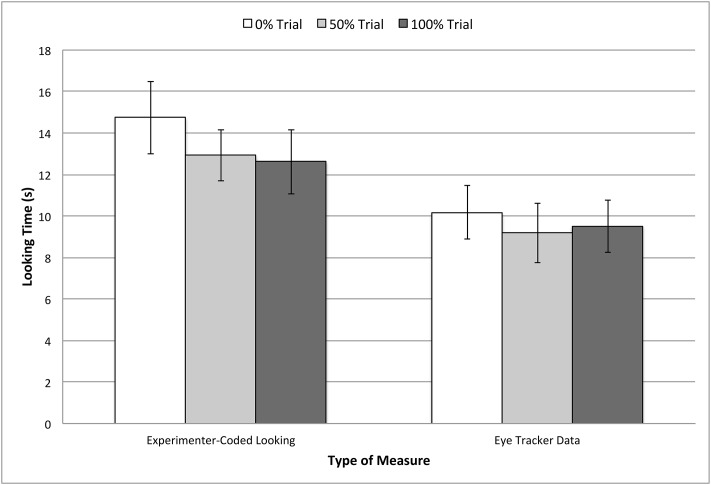
Looking times to the whole scene in familiarization trials. Data are from the critical event in these trials (i.e., revealing the contents of the containers), and plotted separately by the type of measure used (experimenter-coded looking times, or cumulative fixation times to the whole scene as measured by the eye tracker). Error bars represent standard errors.

A repeated-measures analysis of variance (ANOVA) was run on whole scene gaze data during the familiarization trials with a within-subjects factor of TRIAL TYPE (whether the revealed container contained 0%, 50% or 100% of the subsequently sampled color). This factor was not significant, *F*(2, 36) = .32, *p* = .73, suggesting no baseline preferences. To confirm these results, another ANOVA was conducted on looking times coded online by the experimenter, and statistical results were nearly identical, *F*(2, 36) = 1.24, *p* = .30. A final ANOVA with within-subjects factors of MEASURE TYPE (eye-tracking or experimenter-coded) and TRIAL TYPE (the target was the 0%, 50%, or 100% container) confirmed that there was no interaction between these two measures, (*F*(2, 36) = 1.12, *p* = .34). However, as shown in Fig 2, overall looking times were higher when coded by the experimenter, *M* = 13.43 s, *SD* = 1.28 s, than when captured by the eye tracker, *M* = 9.63 s, *SD* = 1.11 s, *t*(18) = 6.46, *p* < .001, *d* = 3.17.

#### Test trials

**Fig 3:**
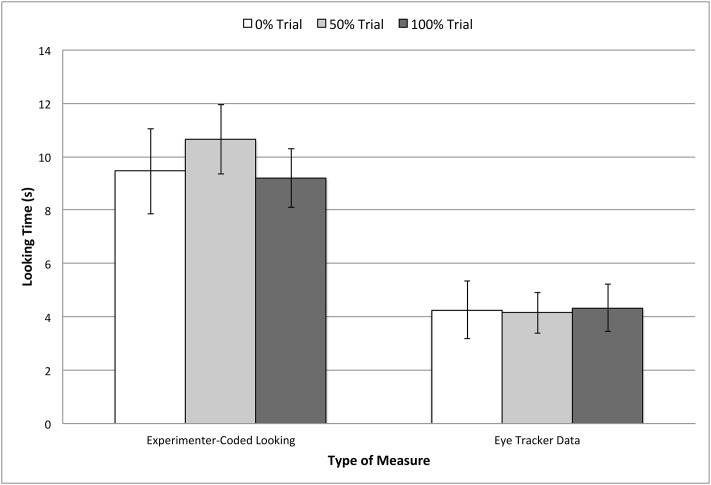
Looking times to the whole scene in test trials. Data are from the critical event in these trials (i.e., after sampling the containers), and plotted separately by the type of measure used (experimenter-coded looking times, or cumulative fixation times to the whole scene as measured by the eye tracker). Error bars represent standard errors.

The critical analysis was a repeated-measures ANOVA on whole scene gaze data from the test phase with a within-subjects factor of TRIAL TYPE (whether the sampled container held 0%, 50%, or 100% of the sampled ball). No significant effect was found, *F*(2, 36) = .015, *p* = .99, indicating no sensitivity to the differences in sampling events. To confirm these results, another ANOVA was conducted on looking times coded online by the experimenter, and statistical results were again nearly identical, *F*(2, 36) = .61, *p* = .55. A final ANOVA with within-subjects factors of MEASURE TYPE (eye-tracking or experimenter-coded) and TRIAL TYPE (the target was the 0%, 50%, or 100% container), which confirmed again that there was no interaction between these two measures, (*F*(2, 36) = .96, *p* = .39). As seen in the familiarization phase, overall looking times were higher when coded online by the experimenter, *M* = 9.77 s, *SD* = 1.09 s, than when captured by the eye tracker, *M* = 4.24 s, *SD* = .68 s, *t*(18) = 6.02, *p* < .001, *d* = 6.09. Overall results, shown in Fig 3, indicated that gaze to the whole screen did not vary as a function of the likelihood of the sampling event in the test trials. This was true for both eye tracker as well as experimenter-coded measures of whole scene looking.

### Infants’ gaze to the areas of interest (AOIs)

Next we tested the possibility that looking at specific areas in the scene would be more informative than just to the whole scene. As mentioned above, the three AOIs were defined over each large container and its associated small container (see Fig 1). Note that this analysis looked at cumulative gaze to the AOIs, but an additional analysis looking at gaze to the AOIs as a proportion measure is in the Supporting Materials (S1 Text, S1 and S2 Figs), and showed similar results.

#### Familiarization trials

To measure possible baseline preferences for the target containers, a repeated-measures ANOVA was run on data from the familiarization phase with within-subjects factors of TRIAL TYPE (whether the revealed container contained 0%, 50% or 100% of the later sampled color) and AOI (defined over the 0%, 50%, or 100% containers). Mauchly’s test showed that the assumption of sphericity was violated for the interaction, *χ*^*2*^(9) = 49.01, *p* < .001, and so degrees of freedom were corrected using Greenhouse-Geisser estimates (*ε* = .55). The interaction was significant, *F*(2.19, 39.41) = 47.14, *p* < .001, *η*_*p*_^*2*^ = .72 and no other reliable effects were seen in the ANOVA (all other *p*’s > .34).

**Fig 4:**
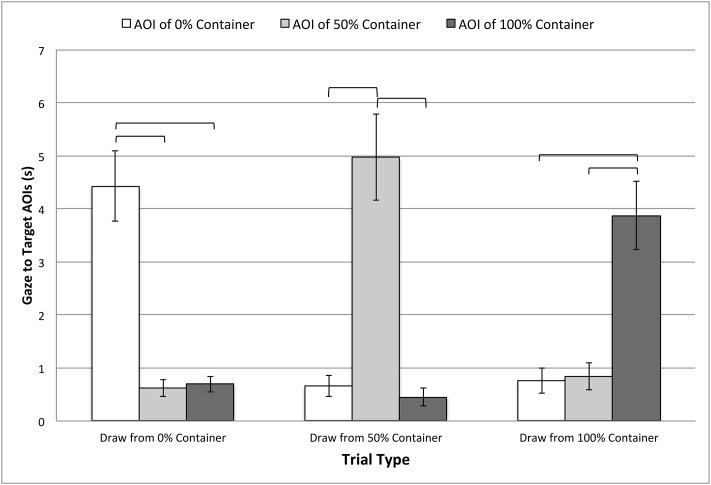
Gaze to the target AOIs in familiarization trials. Data are cumulative fixation times to the target AOIs during the critical event in these trials (i.e., revealing the contents of the containers), and plotted separately by trial type. Error bars represent standard errors, and solid brackets indicate significant differences (Bonferroni-corrected *alpha* = .017).

Planned comparisons similarly showed violations of sphericity (all *p*’s < .005), and so Greenhouse-Geisser corrections were again used (*ε*’s = .55 –.68). All comparisons still showed that looking to the AOIs differed by trial type: When the 0% container was revealed, *F*(1.10, 19.76) = 33.38, *p* < .001, *η*_*p*_^*2*^ = .65; when the 50% container was revealed, *F*(1.10, 19.77) = 35.16, *p* < .001, *η*_*p*_^*2*^ = .66; and when the 100% container was revealed, *F*(1.37, 24.57) = 25.23, *p* < .001, *η*_*p*_^*2*^ = .58. Post-hoc comparisons, summarized in Fig 4, indicated that infants consistently looked longer at the target AOI (the one that included the revealed container) in all conditions. This result validates the utility of AOIs to capturing interest in the target containers.

#### Test trials

The critical analysis was a repeated-measures ANOVA on gaze during test trials with within-subjects factors of TRIAL TYPE (whether sampled container held 0%, 50%, or 100% of the sampled ball) and AOI (defined over the 0%, 50%, or 100% containers). Mauchly’s tests again showed that the assumption of sphericity was violated for the interaction, χ^2^(9) = 33.48, *p* < .001, and so degrees of freedom were corrected using Greenhouse-Geisser estimates (ε = .52). Results yielded a significant interaction, *F*(2.08, 37.46) = 10.69, *p* < .001, *η*_*p*_^*2*^ = .37, and no other main effects (all other *p*’s > .11).

**Fig 5:**
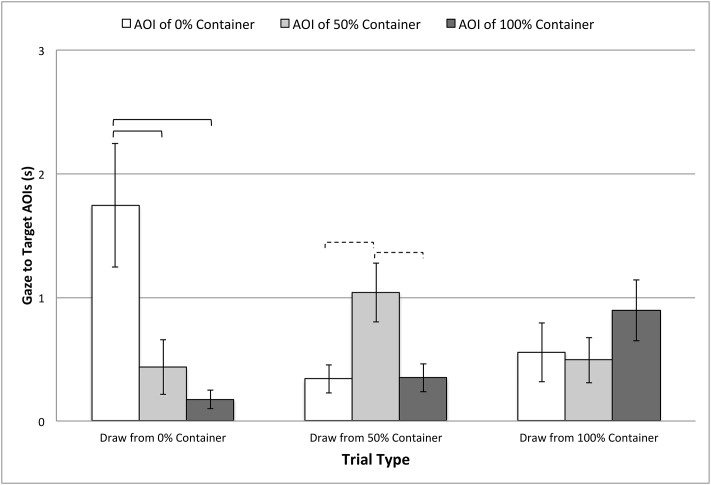
Gaze to the target AOIs in test trials. Data are cumulative fixation times to the target AOIs during the critical event in these trials (i.e., after sampling the containers), and plotted separately by trial type. Error bars represent standard errors. Solid brackets indicate significance with a Bonferroni correction (*alpha* = .017), while dashed brackets indicate significance at *alpha* = .05.

Two of the three planned comparisons showed violations of sphericity^*2*^ (both *p*’s < .004), and so Greenhouse-Geisser corrections to the degrees of freedom were used in both cases (*ε*’s = .65 –.68). Overall, results showed that looking to the AOIs differed for only two trial types: When the 0% container was sampled: *F*(1.30, 23.41) = 8.72, *p* = .001, *η*_*p*_^*2*^ = .33; when the 50% container was sampled: *F*(2, 36) = 7.97, *p* = .001, *η*_*p*_^*2*^ = .31. However, when the 100% container was sampled, looking to the AOIs did not significantly differ: *F*(1.36, 24.38) = 1.85, *p* = .19. Post-hoc comparisons, summarized in Fig 5, showed that infants consistently looked longer at the target AOI (the sampled container) in the 0% sampling event. In the 50% sampling event, looking was longer to the target AOI, but this effect was less reliable. In the 100% sampling event, looking did not significantly differ across AOIs.

## Discussion

Many studies of cognitive ability in infancy depend on whole-scene LTs, including research on infants’ inferential reasoning [7–9; 12–14]. While this method is usually robust, recent findings [10,11] have shown that LTs in infants 8 to 12 months of age do not reflect inferential reasoning under particular circumstances, for example, when single samples are drawn from large populations. This conflicts with studies using other measures, which report successful reasoning abilities under those exact circumstances, and at similar ages [30,31].

This is suggestive of task-dependent discrepancies in infants’ interpretation of sampling events, and as a first step towards investigating this hypothesis, we tested 8 to 11-month-old infants using an eye tracker. In a simplified paradigm, we presented sampling events that were either deterministic or neutral: Infants saw a ball drawn from containers containing 0%, 50%, or 100% identically colored balls. As predicted, our analysis of looking times to even *more* contrastive deterministic events replicated the null results previously found in the probabilistic sampling literature.

Strikingly, an analysis of *where* infants gazed after the sampling event yielded a very different pattern of results. Infants observing the most surprising event (sampling from the 0% container) showed increased gaze to the sampled container and the sample itself, compared to the other containers. This was also true when sampling from the 50% container, but results were less strong, likely due to the statistically neutral ratio. No reliable differences were seen in gaze when the 100% container was sampled.

What does this result—the absence of an effect from standard LT and increased attention to critical regions following surprising sampling outcomes—tell us about infants’ processing of these events? One possibility is that the magnitude of infants’ surprise in these unexpected single event sampling cases is not strong enough to provoke a typical violation of expectation LT response, but that these unexpected events still pique their curiosity. That is, they have noticed that the event was unusual and they are motivated to explore it further. What precisely are infants searching for when looking at the specific locations of unexpected events? One possibility is that infants look for potential causes when unexpected samples are observed (e.g., an unusual property of the box, or of the sample itself). Because infants are sensitive to the physical properties of containers when they are presented in plain view [8], it seems reasonable that our participants might have actively searched for such physical constraints when viewing surprising sampling outcomes.

A corollary of this interpretation is that infants are drawn to other parts of the visual scene when expected events occur, and are relatively less interested in examining the source of the sample. While it remains unclear what infants were fixating in the trials where there was no, or very little surprising information (i.e., the 100% trials, and the 50% trials), it is important to remember that global looking times were not significantly different across conditions. One possibility is that, after expected outcomes, infants spent relatively more time looking at other aspects of the scene (e.g., searching for the woman who had hid herself). Alternatively, infants may simply have scanned randomly when the sampling event was expected. While our current experimental design does not disentangle these possibilities, future work will benefit by measuring infants’ gaze in more diverse experimental contexts, including ones with probabilistic containers. Future work will also benefit from extending this methodology to other questions in infant cognition. Our results suggest that when designing experiments using complex scenes with many potential locations at which to gaze, researchers should take care to assess whether looking to critical regions might reveal an effect otherwise “washed out” in whole scene analyses.

While our study design did not permit an analysis of other data accessible in eye tracking, such as changes in pupil dilation (luminosity was not controlled across trial types), it should be noted that pupillometry is an excellent candidate method to pair with whole-scene LTs in ‘violation-of-expectancy’ paradigms. Both measures can be collected simultaneously, but pupillometry gives more fine-grained, time-locked reactions from individual infants (see also [20]). Future studies should consider designs that allow for the analysis of such measures, which may further shed light upon infants’ precise reasoning processes.

Overall, our results fit with previous literature, which has measured infants’ anticipatory looking to specific locations in a visual scene to test their knowledge of others’ beliefs [7–10], or to test perceptual categorization, among other perceptual and cognitive abilities [11–15]. Our results extend these findings by showing that the location of eye gaze—even *after* an event has occurred (and not just the anticipation of an event)—can reveal something about infants’ reasoning regarding a sampling event’s likelihood (100%, 50%, or 0%). Future research will need to determine when location-specific gaze is necessary, as a wide swath of previous work has found *positive* results using whole scene looking times, and it may be the case that location-specific measures are necessary only when sensitivity to some perceptual or cognitive distinction is too subtle to see otherwise.

One previous result that falls within this line of inquiry has explored infants’ processing of goal-directed actions [35], but their results diverge in an interesting way from ours. Specifically, these researchers suggested that post-hoc looking times to specific locations were *less* sensitive than anticipatory looking times to those locations. Those results are hard to compare with the present ones, as our paradigm likely assesses different cognitive abilities, and uses a very different procedure. Nevertheless, future research must consider what role predictive and post-dictive information plays in guiding eye gaze when perceiving everyday events.

### Conclusion

We replicated previous failures to detect differences in LTs to surprising or unsurprising sampling events [10,11], but our results using an eye tracker showed more fine-grained looking is in fact sensitive to the difference between surprising and unsurprising outcomes. Our results suggest a new interpretation of the divergent findings in the infant reasoning literature. Specifically, looking times in single sampling events (from large populations) may be sensitive to relative likelihood (i.e., converging with previous reports using search measures with probabilistic containers [16]), but only when considering gaze to specific locations. We are currently investigating this hypothesis in an eye tracking procedure that uses probabilistic, rather than deterministic sampling.

Future experimentation must determine why traditional LT measures do not reveal inferential competence in certain paradigms (single objects sampled over large populations), but succeed in others. For example, do crawling paradigms (e.g., [16,17]) capitalize on infants’ motivation to search for desired objects in ways that are distinct from LT paradigms? How might various measures of LT—compared to search paradigms, for example—tap inferences that are either predictive or post-dictive (e.g., [35])? More broadly, researchers should be aware of the complexity of scenes in studies of infant cognition using dynamic events, and consider whether gaze data to critical regions of interest at particular time points could be informative.

## Supporting Information

S1 Fig Proportion of gaze to the target AOIs in the familiarization trials. Data are the proportions of time (relative to total cumulative fixations to the whole screen) that infants spent in the target AOIs during the critical event in these trials (i.e., after revealing the containers), plotted separately by trial type. Error bars represent standard errors. Solid brackets indicate significance with a Bonferroni correction (*alpha* = .017).
(PDF)

S2 Fig Proportion of gaze to the target AOIs in the test trials. Plotted here is the proportion of time (relative to total cumulative fixations to the whole screen) that infants spent in the target AOIs during the critical event in these trials (i.e., after sampling the containers), plotted separately by trial type. Error bars represent standard errors. Solid brackets indicate significance with a Bonferroni correction (*alpha* = .017), while dashed brackets indicate significance at *alpha* = .05.
(PDF)

S1 Text Infants’ gaze to the areas of interest (AOIs): A re-analysis using proportion measures. (DOCX)
