# Supplementary figures and images for: Infants’ Looking to Surprising Events: When Eye-Tracking Reveals More than Looking Time
Source: PLoS One. 2016 Dec 7;11(12):e0164277. doi: 10.1371/journal.pone.0164277 (PMC5142767; doi:10.1371/journal.pone.0164277)

□ 0% Container    ■ 50% Container    ■ 100% Container

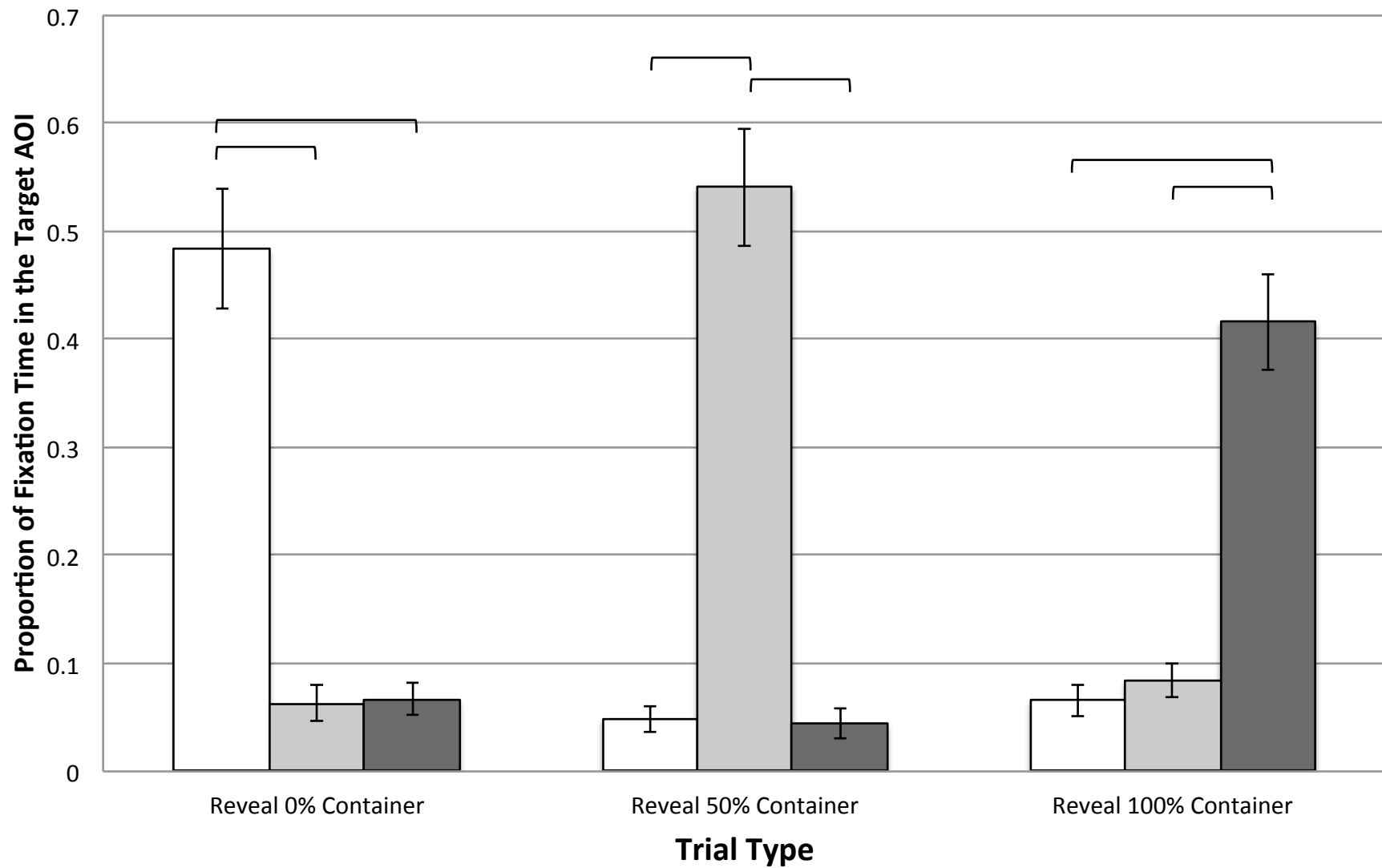

Supplement: S1 Fig — Data are the proportions of time (relative to total cumulative fixations to the whole screen) that infants spent in the target AOIs during the critical event in these trials (i.e., after revealing the containers), plotted separately by trial type. Error bars represent standard errors. Solid brackets indicate significance with a Bonferroni correction (alpha = .017). (PDF) [file pone.0164277.s001.pdf]

□ 0% Container    ■ 50% Container    ■ 100% Container

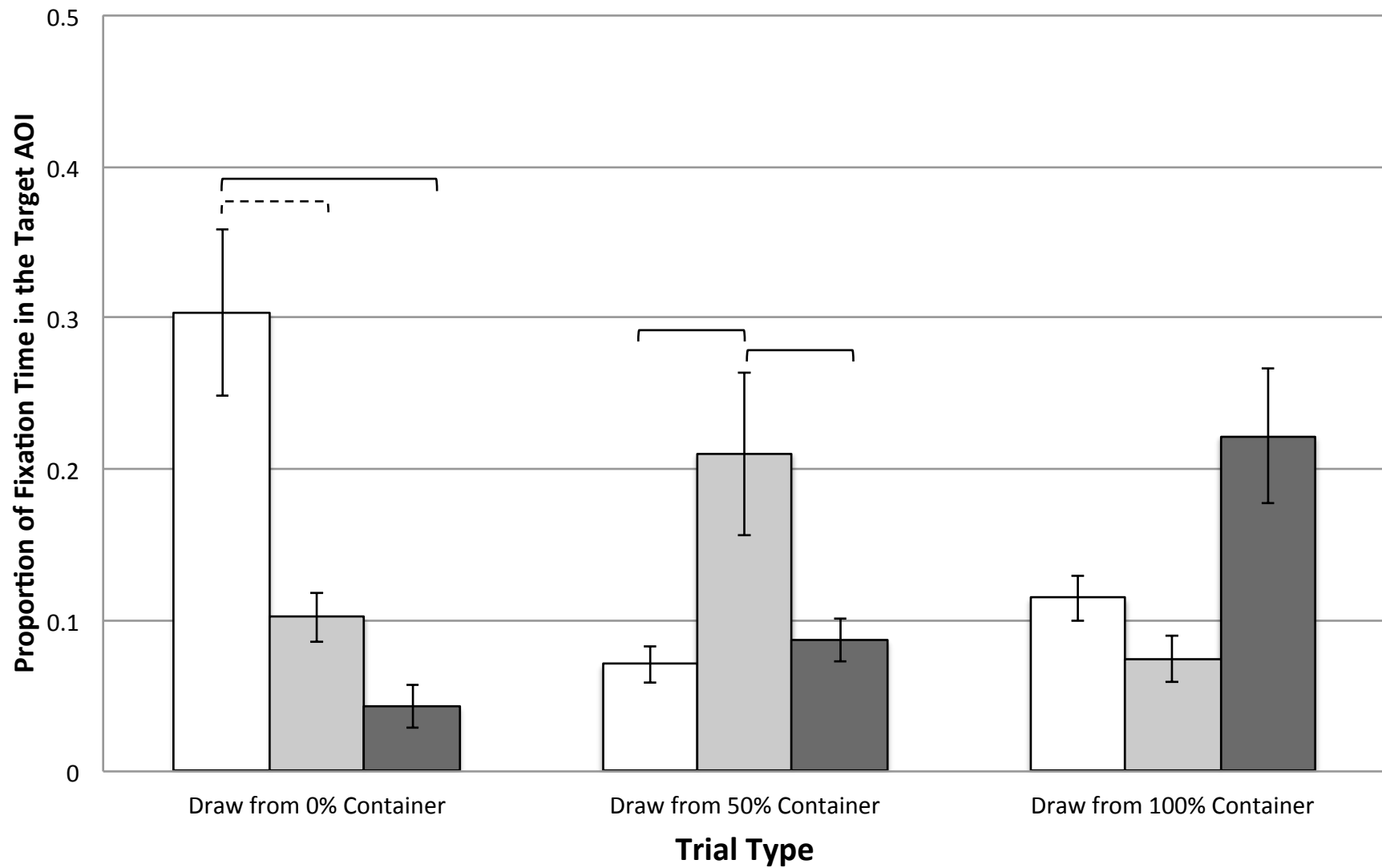

Supplement: S2 Fig — Plotted here is the proportion of time (relative to total cumulative fixations to the whole screen) that infants spent in the target AOIs during the critical event in these trials (i.e., after sampling the containers), plotted separately by trial type. Error bars represent standard errors. Solid brackets indicate significance with a Bonferroni correction (alpha = .017), while dashed brackets indicate significance at alpha = .05. (PDF) [file pone.0164277.s002.pdf]
