# Supplementary material for: Infants’ Looking to Surprising Events: When Eye-Tracking Reveals More than Looking Time
Source: PLoS One. 2016 Dec 7;11(12):e0164277. doi: 10.1371/journal.pone.0164277 (PMC5142767; doi:10.1371/journal.pone.0164277)
Supplement: S1 Text — (DOCX) [file pone.0164277.s003.docx]

**Infants’ gaze to the areas of interest (AOIs): A re-analysis using proportion measures**

Here we re-analyze the AOI data, but instead consider the *proportion* of time that infants spent in each AOI, as a function of their total looking to the screen. This analysis normalizes over different trial lengths, which is an inherent part of infant-controlled procedures, but is not always used in eye-tracking paradigms. The AOIs were defined over the same areas as described in the main text.

**Familiarization trials**

A 3 x 3 repeated-measures ANOVA was run on the proportion of looking to each AOI (relative to the whole screen) during the familiarization phase with within-subjects factors of TRIAL TYPE and AOI. Mauchly’s test showed that the assumption of sphericity was violated for the interaction, *χ^2^*(9) = 52.58, *p* < .001, and the main effect of AOI, *χ^2^*(9) = 6.54, *p* = .038, so degrees of freedom were corrected using Greenhouse-Geisser estimates (*ε* = .48-.76). The interaction was significant, *F*(1.93, 34.69) = 70.03, *p* < .001, *η_p_^2^* = .80, and no other reliable effects, although there was a marginal effect of AOI, *F*(1.52, 27.28) = 2.83, *p* = .089, *η_p_^2^* = .14, due to a tendency to look more at the 50% AOI, which was always located in the middle of the screen.

Planned comparisons similarly showed violations of sphericity (all *p*’s < .005), and so Greenhouse-Geisser corrections were again used (*ε*’s = .55 – .61). All comparisons still showed that looking to the AOIs differed by trial type: When the 0% container was revealed, *F*(1.11, 19.89) = 45.05, *p* < .001, *η_p_^2^* = .72; when the 50% container was revealed, *F*(1.16, 20.82) = 71.27, *p* < .001, *η_p_^2^* = .80; and when the 100% container was revealed, *F*(1.22, 22.03) = 45.77, *p* < .001, *η_p_^2^* = .72. Post-hoc comparisons, summarized in S1 Fig, indicated that infants consistently looked longer at the target AOI (the one that included the revealed container) in all conditions. This result validates the analysis presented in the main text.

**Test trials**

A 3 x 3 repeated-measures ANOVA on the proportion of cumulative fixation time in each AOI was run with within-subjects factors of TRIAL TYPE and AOI. Mauchly’s tests again showed that the assumption of sphericity was violated for the interaction, χ^2^(9) = 18.06, *p* = .035, and so degrees of freedom were corrected using Greenhouse-Geisser estimates (ε = .68). Results yielded a significant interaction, *F*(2.73, 49.12) = 7.76, *p* < .001, *η_p_^2^* = .30, and no other main effects (all other *p*’s > .55).

Just as in the main analysis, planned comparisons were conducted for each trial type (i.e., where each box was sampled). In only one planned comparison (when the 100% box was sampled) was there a significant violation of sphericity according to Mauchly’s test (*p* = .040), and so Greenhouse-Geisser corrections to the degrees of freedom were used in that case (*ε* = .76). Overall, results showed that looking to the AOIs differed for only two trial types: When the 0% container was sampled: *F*(2, 36) = 6.67, *p =* .003, *η_p_^2^* = .27; and when the 50% container was sampled: *F*(2, 36) = 7.12, *p* = .002, *η_p_^2^* = .28. However, when the 100% container was sampled, looking to the AOIs did not significantly differ: *F*(1.52, 27.38) = 1.97, *p* = .17.

Post-hoc comparisons, summarized in S2 Fig, showed that infants looked longer (or marginally longer) at the target AOI containing the sampled container in the 0% sampling event. In the 50% sampling event, looking was also longer to the target AOI. In the 100% sampling event, looking did not significantly differ across AOIs. Just as for the familiarization trials, an analysis over proportion of looking to the AOIs revealed a pattern of results remarkably similar to the analysis in the main text, which examines the cumulative fixation times to the different AOIs.
